# Predictive factors for hypothyroidy after hemithyroidectomy

**DOI:** 10.12688/f1000research.127367.1

**Published:** 2022-11-21

**Authors:** Mohamed Amine Chaabouni, Moncef Sellami, Esma Jameleddine, Rania Kharrat, Wadii Thabet, Malek Mnejja, Boutheina Hammami, Sirine Ayadi, Imen Achour, Ilhem Charfeddine

**Affiliations:** 1 Department of Otorhinolaryngology Head and Neck Surgery, Habib Bourguiba University Hospital, Sfax, Tunisia; 2 University of Sfax, Sfax, Tunisia

**Keywords:** Hypothyroidism, hemithyroidectomy, risk factors, thyroiditis

## Abstract

**Background:** Hemithyroidectomy is one of the most common procedures performed. It is used to treat patients with benign unilateral nodules. Hemithyroidectomy results in fewer risks of hypothyroidism and the need for thyroid hormone replacement therapy. The present study was designed to identify potential clinicopathologic risk factors associated with the onset of biochemical hypothyroidism.

**Methods:** We conducted a retrospective review of all patients who underwent hemithyroidectomy between 2004 and 2019. Hypothyroidism was defined as a serum thyrotropin level greater than 5 mIU/L. The patients were analyzed for age, sex, preoperative and postoperative thyroid stimulating hormone (TSH), state, side, and volume of the remaining lobe, and histologic diagnosis.

**Results:** Hypothyroidism was diagnosed in 30.8% of 214 patients. This complication appeared in the first year in 83.3% of the cases. A preoperative TSH level greater than 1.32 mIU/l, a remaining volume of the lobe less than 3 ml, and the presence of thyroiditis were associated with a significant increase in the risk of developing hypothyroidism (p<0.01). There were no significant differences in age, sex, state, and side of the remaining lobe. The mean thyroxine dose was 57 ± 26 micrograms.

**Conclusions:** The risk of hypothyroidism after hemithyroidectomy should be assessed prior to surgery. Close monitoring is recommended in patients at high risk of developing this complication. However, all patients who undergo hemithyroidectomy should be monitored at least for the first year.

## Introduction

Thyroid lobectomy is one of the most common procedures performed. It is used to treat patients with benign unilateral nodules and may be considered sufficient in some cases of patients with unifocal subcentimeter papillary carcinoma without a significant history of risk factors. ^1^ Thyroid lobectomy also helps prevent the risk of hypocalcemia and bilateral recurrent laryngeal nerve paralysis. ^1^^,^^2^ Furthermore, it was recognized that, by maintaining functioning thyroid tissue, lobectomy results in fewer risks of hypothyroidism and the need for thyroid hormone replacement therapy. ^2^ For these patients, it was common to start prophylactic suppressive hormonal therapy to prevent the occurrence of a nodular recurrence in the remaining lobe. However, the efficiency of this treatment has come under question, especially since it has significant side effects such as atrial fibrillation and osteoporosis, especially in menopausal women. ^3^ Since leaving this therapy, many cases of hypothyroidism have developed after thyroid lobectomy.

Hypothyroidism is an underrated but important complication of thyroid lobectomy with life-threatening side effects. Furthermore, a small percentage of patients on hormone replacement therapy have also been found to continue to complain of fatigue, lack of energy, discrete cognitive disorders, and mood disturbances, despite biochemical euthyroidism. ^1^

Therefore, it is important to determine the risk factors for developing this complication in patients who undergo lobectomy. It will be useful to surgeons, as they will be able to inform patients about this risk, the need for close monitoring, and its duration. ^2^^,^^4^ For example, earlier initiation of thyroid hormone replacement therapy may be recommended for high-risk patients. ^4^ Furthermore, a patient with a small nodule (<1cm) in the remaining gland that will be monitored but with a high risk of developing hypothyroidism after lobectomy, the optimal approach would be total thyroidectomy. ^4^

The purpose of our study was to evaluate the incidence of hypothyroidism after hemithyroidectomy in our patients and to identify risk factors for the development of this complication.

## Methods

### Study design

We conducted a retrospective study of patients who underwent hemithyroidectomy in our department over 15 years (from 2004 to 2019). During this period, preoperative and postoperative TSH were systematically performed.

Hemithyroidectomy was defined as unilateral thyroid lobectomy with or without ismethectomy with preservation of the contralateral lobe.

Inclusion criteria were normal thyroid function during the preoperative period (normal level of TSH), no preoperative thyrotoxin treatment or any thyroid medications were taken, no systematically administered thyroid hormone replacement after the operation, and at least one postoperative measurement of TSH was required.

Patients in whom we discovered thyroid cancer on the definitive histological examination were also excluded from the study.

Postoperative hypothyroidism was defined by a TSH level > 5 mUI/L at any time during the postoperative period. The normal range of TSH in our Hospital laboratory is 0.25–5 mIU/L.

We calculated the remaining volume of the gland considering that the lobe has a roughly ellipsoidal shape. A mathematical formula was adopted: Volume = π/6 × (a × b × c) with a, b, and c representing, respectively, the length, width, and depth determined by ultrasound.

We studied age, sex, preoperative and postoperative TSH levels, operated side, definitive histopathological examination, state of the remaining lobe, follow-up time and the average maintenance dose of Thyroxin for patients who needed thyroid hormone replacement anytime during monitoring.

We used the receiver operating characteristic (ROC) curve to identify the threshold values of the preoperative TSH level and the volume of the remaining lobe that have the best sensitivity-to-sensitivity ratio to predict postoperative hypothyroidism.

### Analysis

Statistical analysis was done using SPSS 20. We performed the Chi-square test for qualitative variables and the Student t test for quantitative variables. The Mann-Whitney test was used for nonparametric variables.

To calculate the risk of occurrence of hypothyroidism over time, we use the Kaplan-Meier method.

A p-value < 0.05 was considered statistically significant.

### Ethical considerations

This study received ethics approval from the Research Ethics Committee of the University Hospital (dated 24 October 2022, approval number 06/2022). In our institution, a retrospective study does not require ethics committee approval. We submitted the study to the ethics committee because it was a requirement of the journal. Written informed consent for publication of their clinical details was obtained from the patients. Written consent to anonymously use patient data for scientific purposes is given by all patients admitted to our department.

## Results

A total of 232 patients were included. ^21^ Eighteen patients were withdrawn from the study as thyroid cancer was discovered on the definitive histological examination. Of 214 patients studied, 66 (30.8%) developed postoperative hypothyroidism.

The mean age in the group of postoperative euthyroid patients and the group of postoperative hypothyroidism was, respectively, 44±13 years and 43±17 years (p=0.92).

The male-to-female sex ratio of the postoperative euthyroid group and the hypothyroidism group was, respectively, 0.09 and 0.06 (p=0.49).

**Figure 1.**
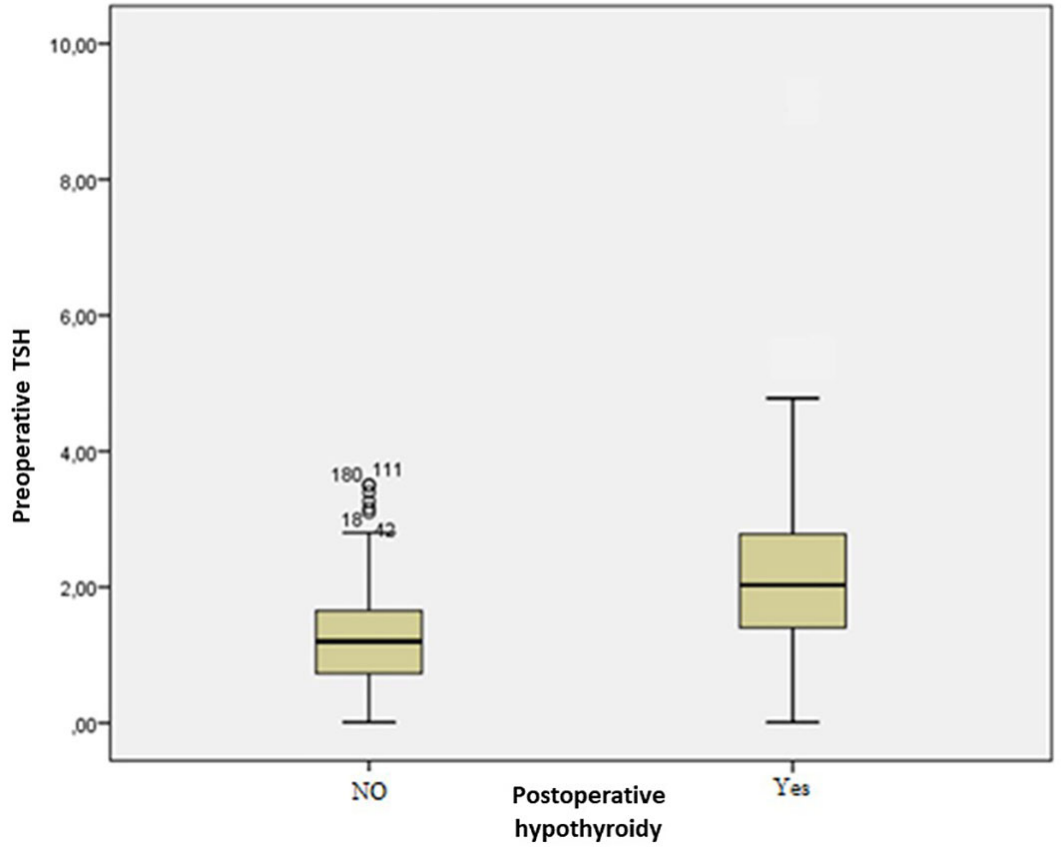
Distribution of patients according to preoperative thyroid stimulating hormone (TSH) levels.

The mean preoperative TSH level for the euthyroid group and the hypothyroidism group was, respectively, 1.2±0.7 and 2.2±1.3 mUI/L (p<0.001) ( Figure 1).

The threshold value of the preoperative TSH level that has the best sensibility-to-sensitivity ratio was 1.32 mUI/L with a sensitivity of 82% and a sensibility of 62%. The risk of developing postoperative hypothyroidism was statistically higher in the group of patients with a TSH below the limit (p<0.001).

Ultrasonography of the thyroid identified seven cases of the remaining hypertrophic lobe, 37 cases of the remaining nodular lobe, and 170 cases of the remaining lobes free from any anomalies. The state of the remaining lobe had no impact on the occurrence of postoperative hypothyroidism.

The mean volume of the remaining lobe was 4.36±2.3 ml for patients who did not develop postoperative hypothyroidism compared to 3.4±2 ml for those who developed it during follow-up. This difference was statistically significant (p=0.01). According to the ROC curve, we derived a threshold volume for the remaining lobe of 3 mL. This volume has the best sensibility to sensitivity ratio, which were respectively 61.8% and 55%.

In total, 91 patients had a right lobectomy and 123 patients had a left one. However, the operated side was not a predictive factor for the occurrence of postoperative hypothyroidism.

The final histopathological examination of the resected sample showed 51 cases of thyroid adenoma and 159 cases of toxic multinodular goiter. The association with lymphocytic thyroiditis was described in 32 cases. Among these histopathological factors, only the presence of thyroiditis was correlated with the risk of developing hypothyroidism (p=0.001). Patients with associated lymphocytic thyroiditis were three times more likely to develop postoperative hypothyroidism (odd ratio: 3.58).

**Figure 2.**
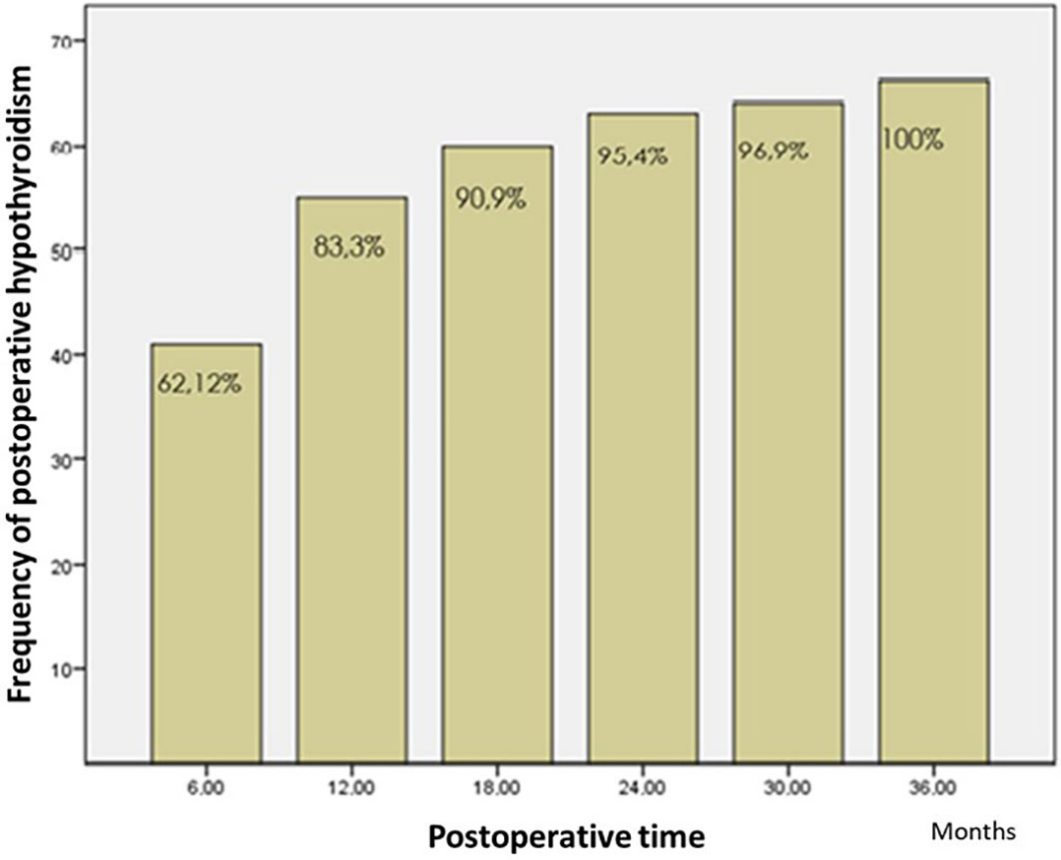
Time to onset of hypothyroidy.

**Figure 3.**
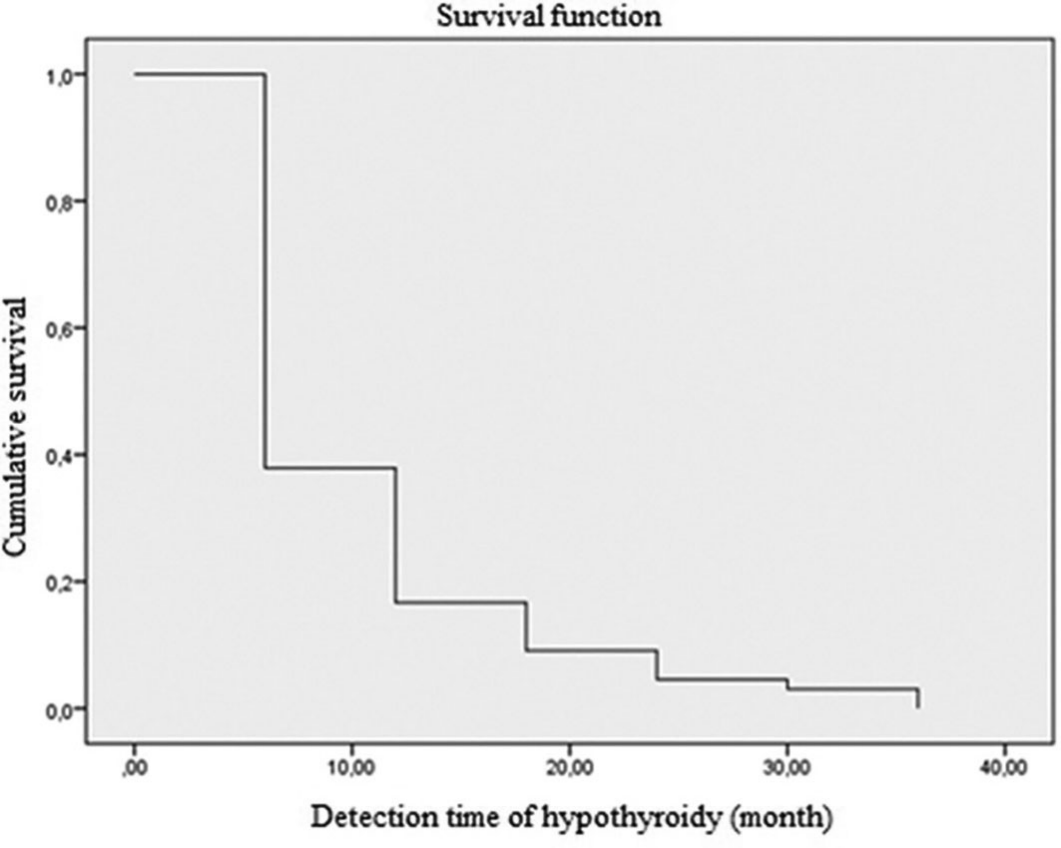
The risk of developing hypothyroidism according to time using the Kaplan-Maier method.

Regarding the time to onset of postoperative hypothyroidism, 62.1% of the patients were diagnosed using laboratory tests in the first 6 months, and 83.3% of the patients within the first year of monitoring ( Figures 2 and 3). The average time to the occurrence of this complication was estimated at 10 months (IC 95% 8.5-12.04).

The mean follow-up duration for the patients was 23 months (4 months – 7 years). Patients who lost follow-up after the first postoperative check-up represented 20.5% of the cases. For patients who had postoperative hypothyroidism, the mean hormonal substitutive dose was 57±26 μg per day.

## Discussion

The half-life of thyroxine produced by the thyroid gland is approximately 7 days. It is recommended to wait at least four to five half-lives of TSH before measuring the TSH level to obtain a precise evaluation of the thyroid hormone produced by the residual thyroid lobe. ^4^ During postoperative monitoring, some patients continue to have normal thyroid function, while in others we noticed an increase in TSH levels. Hypothyroidism can be subclinical with mild TSH levels (TSH from 4.5 to 10 mUI/L) and show a spontaneous return to the euthyroid state due to a compensatory response of the hypothalamic-pituitary-thyroid axis. For patients with a TSH level of less than 10 mUI/L, routine thyroid hormone replacement is not recommended. ^5^ Transient hypothyroidism was found in 33.7 to 67% of the patients. The recovery duration ranges according to the literature from 12 to 18 months. ^6^

Hypothyroidism is one of the most important complications of hemithyroidectomy. Its incidence varies between 6 and 50 % depending on the study. ^2^^,^^7^ This wide range of incidence can be partially attributed to the different normal reference levels of TSH. ^1^ In a meta-analysis that included 32 studies (4899 patients), Verloop *et al*. concluded that the risk of developing postoperative hypothyroidy was approximately 22%. A large proportion of those had subclinical hypothyroidism and one in 25 patients developed clinical hypothyroidism. ^7^ In our study, 30.8% of the patients developed biological postoperative hypothyroidy. However, this complication can be overestimated or underestimated as our mean follow-up duration was 23 months and 20.5% of our patients lost follow-up after their first check-up.

The risk factors attributed to the causation of hypothyroidism in various studies include advanced age, an elevated preoperative TSH level or a normal or high normal value, preoperative hyperthyroidism, autoimmune thyroiditis, multinodular goiter and remnant thyroid volume inferior to 3.2 ml. ^2^^,^^4^^,^^8^

In our study, a preoperative TSH level greater than 1.32 mUI/L was a predictive factor for the development of postoperative hypothyroidism with a sensitivity of 82% and a sensibility of 62%. In a meta-analysis performed in 2013, the authors found that patients with a preoperative TSH level greater than 2.5 μUI/L were three times more likely to develop postoperative hypothyroidism. ^1^ The limit for TSH levels varied from one study to another; 2.6 for Ahn *et al*., ^5^ 2 for Chong *et al*., ^6^ and 1.7 for Park *et al*. ^9^ Said *et al*. found that for each increase in TSH level of 1 μIU/ml, there is an approximate doubling of the risk of hypothyroidism. ^8^ A higher TSH preoperatively in patients undergoing a hemithyroidectomy indicates a lower reserve of thyroid function and tends to develop hypothyroidism. ^2^ Furthermore, for patients with mild hypothyroidism (TSH level <10), the finding of a TSH level per operative at least 2.6 mIU/L was correlated with a higher risk of developing unrecovered subclinical hypothyroidism. ^5^ Park *et al*. found that preoperative serum TSH level >1.7 mIU/L was an independent risk factor for postoperative hypothyroidism and persistent hypothyroidism without recovery. They also found that a 1-year postoperative serum TSH level of at least 3.1 mIU/L was an independent factor in predicting late hypothyroidism during follow-up. ^9^

Furthermore, we have observed that lymphocytic thyroiditis within the resected lobe was associated with a statistically higher risk of developing postoperative hypothyroidism. ^10^ Hashimoto thyroiditis is the most frequent autoimmune disease of the thyroid gland. ^2^ Ahn *et al*. also found in their study a correlation between Hashimoto thyroiditis and the risk of developing postoperative hypothyroidism. ^11^

Lymphocytic infiltration of the thyroid gland can lead to a decrease in its function, and a semiquantitative analysis found that as the degree of lymphocytic infiltration within the resected thyroid gland increased, the possibility of postoperative hypothyroidism also increased. ^12^ Furthermore, according to a review of histological data, postoperative TSH levels were found to be significantly associated with lymphocytic infiltration (LI) and germinal center (GC) formation (considered a histological measure of immunologic activation). Specifically, patients with a high LI/GC score had significantly higher mean TSH levels than those with a low LI/GC score. ^13^

The disadvantage of these scores is their unavailability before the surgery. The level of thyroid antibodies can overcome this problem. Lee *et al* found that the presence of microsomal antibodies was a significant preoperative predictor for levothyroxine supplementation. ^14^ Other studies found a strong association between detectable levels of thyroid antibodies and histological lymphocyte infiltration in the surrounding thyroid gland. ^11^ The measurement of preoperative anti-thyroperoxidase antibodies can be used as a simple tool to estimate the risk of hypothyroidism in more detail before planning surgery. ^7^ However, in our department, the measurement of thyroid antibodies for simple nodules and unilateral goiter in euthyroid patients (both clinical and biological) was not systematic. Therefore, this predictive factor could not be assessed.

Cho *et al*. found that preoperative TSH between 2.0 and 5.9 mIU/L, and two or more positive factors of Thyroglobulin, anti-thyroglobulin, and anti-thyroid peroxidase (anti-TPO) strongly increase the risk of developing postoperative hypothyroidism. ^3^

The residual thyroid volume was evoked as a possible predictive factor for postoperative hypothyroidism. Lang *et al*. found in their prospective study that if the adjusted volume of the body surface area was less than 3.2 ml, the patient is three times more likely to develop this complication. In this same study, the adjusted residual volume of the surface area of the non-body was also a significant risk factor. ^15^ In our study, a residual volume of fewer than 3 ml was correlated with a higher risk of hypothyroidism. Some authors studied the effect of isthmus-preserved thyroid lobectomy and found that the incidence rate of hypothyroidism after this surgery was lower. ^16^

To predict this complication, some authors established a risk-scoring system. One of these scores was established based on preoperative TSH level and age. The incidence of hypothyroidism was 3% with a risk score of 0, 20% with a score 1, 39% with a score 2, and 70% with a score 3. ^17^ This risk scoring system has the advantage of predicting risk before surgery, allowing the surgeon to discuss it with patients.

The time to develop clinical hypothyroidism in our study was 12 months for 83.3% of the patients. This is consistent with most of the studies found in the literature, and most patients develop it at 12 months. ^2^ In a study by Al-Shalhoub *et al*., hypothyroidism was diagnosed in the first 6 months in 70% of patients. ^18^ It appeared in the first 3 months after surgery in 84.5% of cases and after 9 months in 91.2% of patients. ^5^

Identifying risk factors of the need for thyroid hormone supplementation can help surgeons to provide preoperative counseling to patients and to develop a monitoring plan. ^19^

Regarding postoperative monitoring, there is no algorithm or wide consensus on it. However, it is recommended to obtain a first postoperative TSH measurement for all patients who have undergone hemithyroidectomy 3 months after surgery so compensation by the remaining thyroid lobe may occur. ^13^ Some authors suggest that postoperative TSH should be determined at 6 weeks, 6 months, and 12 months postoperatively. ^2^ For others, thyroid function should be evaluated at 3, 6 months, and 1 year after surgery, then once or twice a year thereafter for low-risk patients. Additional monitoring of thyroid function at 2 and 9 months was optional for high-risk patients. ^3^ Seiberling *et al*. believe that it may be wise to follow patients with risk factors (including monitoring thyroid function) more closely during the postoperative period. ^20^ For Park *et al*., patients with high postoperative 1-year TSH levels (3.1 mIU/L) could require more frequent follow-up for thyroid function evaluation, even if they could be euthyroid 1 year after lobectomy. ^9^

## Conclusion

Patients who had a hemithyroidectomy should be closely monitored during the first years after surgery with biological and clinical check-ups one or twice a year. The first follow-up should be 6 months after surgery. The frequency of thyroid function tests and examinations, as well as the proposed timeof the monitoring plan, should be determined depending on the presence or absence of risk factors for developing post-hemithyroidectomy hypothyroidism.

## Data Availability

Figshare: Predictive factors for hypothyroidy after hemithyroidectomy, https://doi.org/10.6084/m9.figshare.21404862.v1. ^21^ This project contains the following underlying data:
- data.sav (SPSS format; all datasets have been de-identified in accordance with the Safe Harbor method.) Data are available under the terms of the Creative Commons Attribution 4.0 International license (CC-BY 4.0).
