# Supplementary material for: Predictive factors for hypothyroidy after hemithyroidectomy
Source: F1000Res. 2022 Dec 21;11:1355. Originally published 2022 Nov 21. [Version 2] doi: 10.12688/f1000research.127367.2 (PMC9811031; doi:10.12688/f1000research.127367.2)
Supplement: Peer review history

# Peer review: Reviewer response for version 2

**DOI:** 10.5256/f1000research.142052.r158507

**Published:** 2023-01-03

**Authors:** Senda Turki

**Affiliations:** 1 Université Tunis El Manar, Tunis, Tunisia

The main points have been taken into account by the authors and corrected.

Is the work clearly and accurately presented and does it cite the current literature?

Partly

If applicable, is the statistical analysis and its interpretation appropriate?

Yes

Are all the source data underlying the results available to ensure full reproducibility?

Yes

Is the study design appropriate and is the work technically sound?

Yes

Are the conclusions drawn adequately supported by the results?

Yes

Are sufficient details of methods and analysis provided to allow replication by others?

Yes

Reviewer Expertise:

Rhinology, Otology, Balance diseases and vertigo, Sleep Apnea, Neck surgery

I confirm that I have read this submission and believe that I have an appropriate level of expertise to confirm that it is of an acceptable scientific standard.

# Peer review: Reviewer response for version 2

**DOI:** 10.5256/f1000research.142052.r158506

**Published:** 2022-12-21

**Authors:** Ingrid Breuskin

**Affiliations:** 1 Department of Head and Neck Oncology, Gustave Roussy Institute, University Paris Saclay, Villejuif, France

Thank you for the modifications.

Is the work clearly and accurately presented and does it cite the current literature?

Partly

If applicable, is the statistical analysis and its interpretation appropriate?

Yes

Are all the source data underlying the results available to ensure full reproducibility?

Yes

Is the study design appropriate and is the work technically sound?

Yes

Are the conclusions drawn adequately supported by the results?

Yes

Are sufficient details of methods and analysis provided to allow replication by others?

Yes

Reviewer Expertise:

head and neck cancer

I confirm that I have read this submission and believe that I have an appropriate level of expertise to confirm that it is of an acceptable scientific standard.

# Peer review: Reviewer response for version 1

**DOI:** 10.5256/f1000research.139867.r156290

**Published:** 2022-12-07

**Authors:** Senda Turki

**Affiliations:** 1 Université Tunis El Manar, Tunis, Tunisia

This article addresses a particularly important topic. Indeed, the risk factors for hypothyroidism after hemithyroidectomy must be evaluated precisely, given the fact that it’s a frequent surgical option for managing several unilateral thyroid diseases. There are several articles about this topic in the literature and different factors have been identified.

 This study is interesting, easy to follow and clear to understand. The series studied is important with a well conducted statistical study.

 However, I have a few comments that the authors might consider:

**1. **The introduction is too long (for example: the part about systematic hormone supplementation should be displaced to the beginning of the discussion).

**2. Methods:**

- “Benign definitive histological examination” should be added to the inclusion criteria. Indeed, patients with papillary cancer aren’t initially included in the study. They aren’t excluded from it. Moreover, why were these patients not included (several low-risk papillary cancers can be treated by hemithyroidectomy)?
- The ROC’s explanation is unclear, especially “the sensitivity-to-sensitivity ratio” which is also mentioned in the results part: “The Receiver operating characteristic (ROC) curves compare sensitivity versus specificity across a range of values for the ability to predict a dichotomous outcome.” (Statistic courses).

**3. Results:**

- The risk of developing postoperative hypothyroidism was higher in the group of patients with a TSH **above** the limit, not below.
- There is no information regarding your patients’ clinical symptoms of hypothyroidism.
- You could have added figures and tables for the description of your series.

**4. Discussion:**

- "In our study, a preoperative TSH level greater than 1.32 mUI/L was a predictive factor for the development of postoperative hypothyroidism with a sensitivity of 82% and a sensibility of 62%.”: do you mean 'specificity' instead of 'sensibility'?
- In the literature, the female sex is also a risk factor.

**5**. You could have added in the reference a recent meta-analysis: Li *et al.* (2020 ^1^).

Is the work clearly and accurately presented and does it cite the current literature?

Partly

If applicable, is the statistical analysis and its interpretation appropriate?

Yes

Are all the source data underlying the results available to ensure full reproducibility?

Yes

Is the study design appropriate and is the work technically sound?

Yes

Are the conclusions drawn adequately supported by the results?

Yes

Are sufficient details of methods and analysis provided to allow replication by others?

Yes

Reviewer Expertise:

Rhinology, Otology, Balance diseases and vertigo, Sleep Apnea, Neck surgery

I confirm that I have read this submission and believe that I have an appropriate level of expertise to confirm that it is of an acceptable scientific standard, however I have significant reservations, as outlined above.

## References

[1] : Prevalence of and risk factors for hypothyroidism after hemithyroidectomy: a systematic review and meta-analysis. *Endocrine* .2020;70(2) : 10.1007/s12020-020-02410-5 243-255 10.1007/s12020-020-02410-5 32638212

# Peer review: Reviewer response for version 1

**DOI:** 10.5256/f1000research.139867.r156287

**Published:** 2022-11-28

**Authors:** Ingrid Breuskin

**Affiliations:** 1 Department of Head and Neck Oncology, Gustave Roussy Institute, University Paris Saclay, Villejuif, France

- This is a study about the predictive factors for hypothyroïdism after loboisthmectomy.
- It shows that preoperative TSH, thyroïditis and the volume of the remaining lobe are predictive.
- It is an interresting work, nicely written and easy to understand.
- It is maybe a bit too descriptive. A table with the different characteristics would be interresting and more visual (age, sex, histological results.)
- It would have also been interesting to analyse what happens after 1 year. As 20% of the patients with hypothyroidism will develop hypothyroidism after this period.
- Why have they excluded patients with cancer? It is because they have all undergone a completion thyroidectomy? If yes ok but if no, they should be integrated.
- In the introduction part, I would not discuss about systematic hormone supplementation for hemithyroidectomy as it is not done anymore, or I would greatly temper the statement.

Is the work clearly and accurately presented and does it cite the current literature?

Partly

If applicable, is the statistical analysis and its interpretation appropriate?

Yes

Are all the source data underlying the results available to ensure full reproducibility?

Yes

Is the study design appropriate and is the work technically sound?

Yes

Are the conclusions drawn adequately supported by the results?

Yes

Are sufficient details of methods and analysis provided to allow replication by others?

Yes

Reviewer Expertise:

head and neck cancer

I confirm that I have read this submission and believe that I have an appropriate level of expertise to confirm that it is of an acceptable scientific standard, however I have significant reservations, as outlined above.
